# Design of thin, wideband electromagnetic absorbers with polarization and angle insensitivity using deep learning

**DOI:** 10.1038/s41598-025-94116-9

**Published:** 2025-03-19

**Authors:** Atefe Shahsavaripour, Mohammad Hossein Badiei, Ahmad Kalhor, Leila Yousefi

**Affiliations:** 1 Electrical and Computer Engineering, College of Engineering, University of Tehran, Tehran, 1417614411 Iran; 2 School of Engineering and Informatics, University of Sussex, Falmer, UK

**Keywords:** Engineering, Electrical and electronic engineering, Applied mathematics

## Abstract

Metamaterial-based electromagnetic absorbers, despite being thin and lightweight, typically suffer from narrow-band frequency bandwidth and sensitivity to polarization and incident angle due to their resonant nature. Previous methods to increase bandwidth have shown improvements but have not fully succeeded in developing wide-band, thin metamaterial-based absorbers suitable for mass production. In this study, we introduce a novel approach that leverages artificial intelligence to design a thin, wideband metamaterial-based absorber covering the entire frequency range of 8-12 GHz. The proposed method utilizes a Generative Adversarial Network (GAN), given the need for precise structural details and computational efficiency, which globally outperform variational autoencoders (VAEs) and diffusion models, for parameter estimation and a Multi-Layer Perceptron (MLP) network as a simulator to predict the electromagnetic response of the designed absorber and provide feedback to the generative network. Numerical full-wave electromagnetic simulations serve as the training data and ground truth for both the GAN and MLP networks. This training enables the generative network to produce structures with high absorption, while the MLP predicts the corresponding absorbance value for each structure. This approach allows for the rapid design of various real-world structures, quick calculation of their absorption values using the MLP network, and selection of the most optimal structures for fabrication. The performance of the designed metamaterial-based absorber is verified both numerically and experimentally. Results show an absorption rate above 90% for all frequencies in the range of 8-12 GHz. The structure also operates effectively for both TE and TM polarizations and for all incident angles between 0-45 degrees. Additionally, the designed structure can be easily fabricated using printed circuit board (PCB) technology, making it practical and suitable for mass production.

## Introduction

Electromagnetic (EM) absorbers are structures designed to attenuate EM radiation across specific frequency ranges by effectively converting incident EM energy into heat. These absorbers are crucial in applications such as electromagnetic interference (EMI) mitigation^1^, energy harvesting^2,3^, and photodetection^4^, where they enhance system performance and sustainability by minimizing unwanted EM reflections and emissions.

Metamaterial-based absorbers offer significant advantages over traditional EM absorbers, including much lower thickness, less weight, and higher flexibility^5–7^. These absorbers are constructed from metamaterials, which are artificially engineered structures with unique electromagnetic properties defined by their sub-wavelength unit cells. This allows for tailored responses to EM waves that cannot be achieved by natural materials^8,9^. However, the narrow-band characteristics of metamaterials significantly restrict the bandwidth of these absorbers making them highly susceptible to frequency deviations^5–10^.

In response to these limitations, recent research has explored strategies to overcome the bandwidth constraints of conventional metamaterial absorbers. To begin with, LPDA-inspired absorbers utilize log-periodic designs to achieve broad absorption performance through multiple resonances across a wide frequency range^11^. However, their reliance on precise fabrication and alignment introduces significant practical challenges. Similarly, Kirigami-inspired metamaterial absorbers leverage mechanical tunability to dynamically enhance absorption characteristics^12^. Yet, their dependence on complex mechanical deformations limits their scalability and long-term stability for real-world applications. The triple-band polarization-insensitive metamaterial transmission line absorber demonstrates effective absorption at three distinct frequency points^13^. However, its functionality is limited to specific frequencies and does not extend to continuous wideband absorption, which is essential for broader EMI suppression and similar applications. Additionally, ultra-broadband absorbers based on advanced nanophotonic designs, such as those presented in a research work based on the square-patch structure loaded with linear circuit components, utilize anisotropic structures and novel material compositions to achieve suitable absorption performance^14^. Despite these advances, the reliance on expensive materials and intricate fabrication techniques poses significant barriers to practical implementation in cost-sensitive and large-scale applications. These shortcomings highlight the need for alternative approaches that combine wideband performance with practical scalability and robust operational stability.

One of the solutions proposed to increase the operational bandwidth of metamaterial-based absorbers is utilization of multi-resonance techniques and novel material compositions^15–17^. Despite these efforts, practical constraints like substrate thickness and non-flat absorption profiles hinder their applications. Some works have sought to enhance bandwidth by integrating lumped circuit elements, but this approach introduces significant fabrication complexities, making the resultant structures impractical for mass production^18^. An example is the multi-mode-assisted broadband impedance-gradient thin metamaterial absorber, which utilizes multi-mode resonance mechanisms to achieve wideband absorption^19^. While this design demonstrates suitable performance in controlled conditions, its dependence on multi-layered configurations and costly impedance-gradient materials imposes significant barriers to practical implementation. Similarly, Ranjan et al. demonstrated a wideband absorber structure through the integration of multi-layered configurations and innovative design principles, yet concerns about substrate thickness persist^20^. Another example is a broadband absorber which employs a combination of multilayered metasurfaces and resonant elements to extend the operational bandwidth^21^. While this approach achieves convincing results in terms of absorption efficiency, the reliance on multiple layers significantly increases the overall thickness and fabrication complexity, limiting its scalability and practical application in cost-sensitive industries.

Recently, there has been growing interest in leveraging deep learning methodologies to define optimal parameters for metamaterial-based design^22–25^. Deep neural networks excel in learning from extensive datasets and discovering intricate electromagnetic response patterns that traditional methods struggle to discern. In this regard, Hou et al. introduced a novel deep learning approach for inverse design of metamaterials with high-absorption materials, which are typically costly and thick^26^. Building on this progress, Zhou et al. proposed an equivalent-circuit-intervened deep learning metasurface framework that incorporates circuit models into deep learning algorithms to enhance the accuracy of electromagnetic response predictions^27^. This hybrid approach effectively bridges the gap between theoretical models and practical implementations, offering improved precision in the design process. Furthermore, Lin et al. advanced the field with a fuzzy inverse design method utilizing a generative adversarial network (GAN) to address the limitations of traditional design methods. However, their approach relies on high-absorption materials, leading to increased design costs and thickness, which pose challenges for the development of low-cost, thin, wide-band metamaterial-based absorbers^28^.

In this work, we have designed, fabricated, and measured a thin, wideband metamaterial-based absorber using generative deep learning techniques. The structure can be easily fabricated using standard PCB techniques and utilizes an inexpensive FR4 substrate, significantly reducing overall costs and making it suitable for mass production. Furthermore, it shows insensitivity to the angle of incidence and polarization of the incident wave, retaining its performance for both TE and TM polarization and all incidence angles in the range of 0 to 45 degrees. Our proposed method employs GANs to optimize design parameters and MLPs to predict precise absorption values, thereby achieving a metamaterial-based absorber with advanced properties. The GAN generates diverse structure configurations, while the MLP quickly predicts their absorption rates, allowing for the selection of the most optimal designs with over 90% absorption. Considering the specific requirements of our metasurface design, such as the need for precise structural details and computational efficiency during inference, GANs are generally preferred over VAEs and diffusion models due to their ability to generate sharp, high-fidelity designs quickly and learn compact latent representations, making them a strong candidate for such design optimization tasks. This deep learning approach significantly accelerates absorber design by drastically reducing simulation time, leading to enhanced performance, broader bandwidth, and improved tolerance to diverse electromagnetic conditions. By integrating the generative network with rapid absorption prediction, the selection of optimal structures for fabrication is streamlined. The numerical and experimental results achieved for the optimal structure illustrate its advantages over previously reported works in this area, as clearly shown in Table 5 at the end of the paper.

## Proposed methodology: generative deep learning-based design

The wideband absorption is well-suited for applications in various domains, including radar systems for stealth technology and radar cross-section (RCS) reduction, EMI mitigation for shielding sensitive electronics^29^, and energy harvesting by capturing ambient electromagnetic energy^30^.

### Geometric configuration of the proposed metamaterial-based absorber

**Fig. 1:**
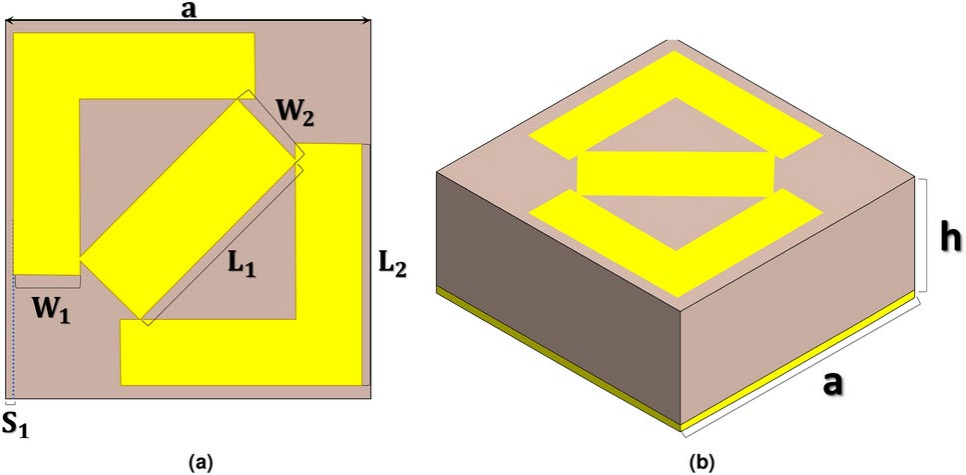
Unit cell of the proposed metamaterial-based absorber (**a**) top view (**b**) perspective.

The proposed metamaterial-based absorber is illustrated in Fig.1 This structure comprises three distinct layers: a resonant top layer, a fully metallic ground plane made of copper, and a dielectric substrate of FR4. The top layer is designed to provide a wide-band absorptive response, while the bottom layer prevents EM waves from escaping the structure. Both the top and bottom layers are made of copper, with a thickness of 35 ${\upmu }$m and a conductivity of $\sigma = 5.96 \times 10^7$ S/m. These layers are separated by a 2 mm thick dielectric substrate with a relative permittivity of $\varepsilon _r = 4.4$, and loss tangent of $tan\delta = 0.02$ . The goal of the proposed design is to shape the top layer to achieve high EM absorption across the entire X-band regime.

**Fig. 2:**
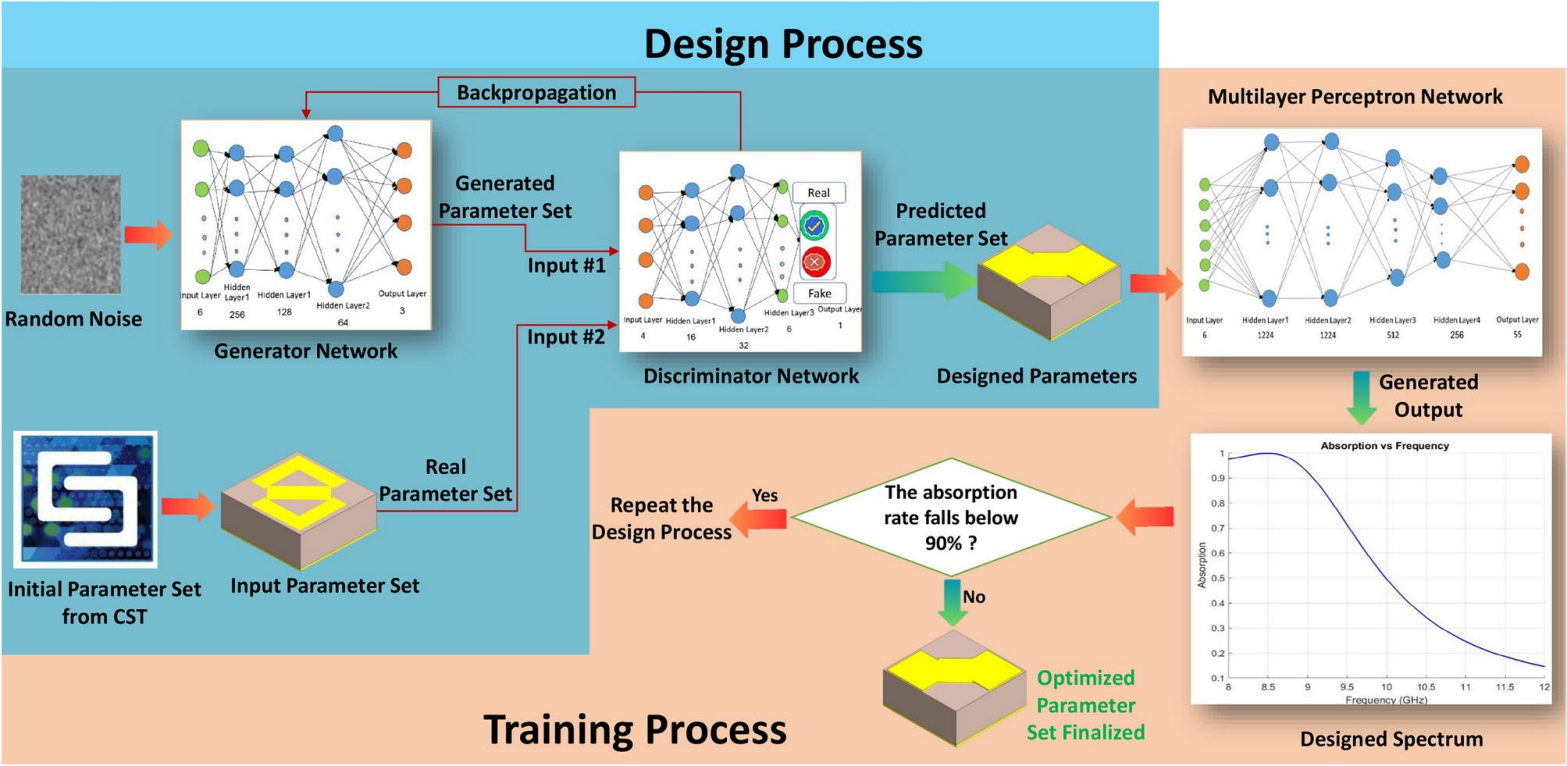
Overview of the proposed methodology.

The proposed design methodology is illustrated in Fig. 2, which depicts the architecture of the developed neural network. The process begins with the training of the generator and discriminator to ensure optimal performance. Once trained, the generator iteratively estimates design parameters for the unit cells to achieve high absorption. Within each iteration, each design is then evaluated by the MLP network, which has been trained to predict absorption values based on the learned relationship between design parameters and their corresponding absorption values. If the predicted absorption values meet the desired criteria, the process concludes. Otherwise, the generator refines the design parameters further, guided by feedback from the MLP predictions. This iterative process continues until an optimal design with satisfactory absorption values is achieved. By combining the generative capabilities of the GAN with the predictive power of the MLP, this methodology effectively narrows down the optimal unit cell design and parameters, ensuring high absorption efficiency across a wide frequency spectrum.

As the first step, we generate a dataset to train the neural network for predicting electromagnetic absorption values. This involves curating diverse structures as inputs and their corresponding absorption values as ground truths. These paired data points form the basis of the neural network’s learning process, enabling fast and precise predictions. Training data is generated through full-wave simulation using the finite integration technique within CST Microwave Studio, employing periodic boundary conditions. The absorption rate $A(\omega )$ is then calculated using numerical data generated in CST as follows:

1 $$\begin{aligned} A(\omega ) = 1 - |S_{11}(\omega )|^2 - |S_{21}(\omega )|^2, \end{aligned}$$

where $S_{21}(\omega )$ is the transmission coefficient and $S_{11}(\omega )$ is the reflection coefficient. As we utilize a fully metallic ground plane in our design, the transmission of incident waves is inhibited so that $S_{21}(\omega ) = 0$.

In the following, we provide detailed explanations for the various components of the architecture illustrated in Fig.2.

### Generative adversarial network for rapid structure estimation

We have designed a GAN to estimate the geometry of the metamaterial top layer that provides higher absorption. GAN structures play a pivotal role in generating synthetic data that is nearly indistinguishable from real data. This framework consists of two neural networks: Generator $(\textbf{G})$ and Discriminator $(\textbf{D})$, which engage in a dynamic process of competition and collaboration.

**Generator network:** The generator network is designed to create synthetic parameters starting from an input noise vector $\textbf{z}$. The vector $\textbf{z}$ is a random noise vector sampled from a predefined probability distribution. Here, we use a standard unimodal noise vector with a dimensionality of 3, balancing the variability of generated design parameters with computational efficiency. Its goal is to produce data $\textbf{G}(\textbf{z}; \theta _g)$ that closely resembles the real design parameters used in our study. Here, $\theta _g$ represents the set of learnable parameters of the generator network, including weights and biases. During training, these parameters $\theta _g$ are iteratively adjusted to minimize the difference between the generated data $\textbf{G}(\textbf{z}; \theta _g)$ and the real design parameters.

**Discriminator network:** The discriminator network assesses the data produced by the generator, distinguishing between real and synthetic input data to refine the generator’s output. We utilize ReLU activation functions in its intermediate layers and a sigmoid activation function in its final layer to output a probability score $\textbf{D}(\textbf{x}; \theta _d)$, where $\theta _d$ represents the learnable parameters of the discriminator network that are optimized during training to improve its classification accuracy.

The interaction between the generator and discriminator networks is a process of mutual refinement: as the generator $\textbf{G}$ improves its ability to create realistic data, the discriminator $\textbf{D}$ simultaneously enhances its capacity to identify synthetic or fake data. The loss functions $\mathscr {L}$ for the generator and discriminator are defined as follows^31^:

2 $$\begin{aligned} \mathscr {L}_\textbf{D}&= -\mathbb {E}_{\textbf{x} \sim p_{\text {data}}} [\log \textbf{D}(\textbf{x})] - \mathbb {E}_{\textbf{z} \sim p_{\textbf{z}}} [\log (1 - \textbf{D}(\textbf{G}(\textbf{z})))], \end{aligned}$$

3 $$\begin{aligned} \mathscr {L}_\textbf{G}&= -\mathbb {E}_{\textbf{z} \sim p_{\textbf{z}}} [\log \textbf{D}(\textbf{G}(\textbf{z}))]. \end{aligned}$$

Here, $\mathbb {E}$ represents the expected value, $p_{\text {data}}(\textbf{x})$ is the real data distribution, and $p_{\textbf{z}}$ denotes the input noise distribution.

As shown in Fig. 2, the generative network is employed for the fast and efficient design and parameter tuning of unit cells. Within this architecture, the generator produces synthetic unit cell designs, while the discriminator assesses them against real designs. This interaction between the networks facilitates the generation of highly optimized unit cell structures. The specific details of the generator and discriminator networks used in our approach are summarized in Table 1.

**Table 1:** Details of the generator and discriminator networks.

Network	Layer Type	Details
Generator	Input Layer	Input Size: 3
	Dense Layer	Units: 16, Activation: ReLU
	Dense Layer	Units: 32, Activation: ReLU
	Output Layer	Units: 6
Discriminator	Input Layer	Input Size: 6
	Dropout Layer	Dropout Rate: 0.3
	Dense Layer	Units: 256, Activation: ReLU
	Dropout Layer	Dropout Rate: 0.3
	Dense Layer	Units: 128, Activation: ReLU
	Dropout Layer	Dropout Rate: 0.3
	Dense Layer	Units: 64, Activation: ReLU
	Output Layer	Units: 3, Activation: Sigmoid

**Fig. 3:**
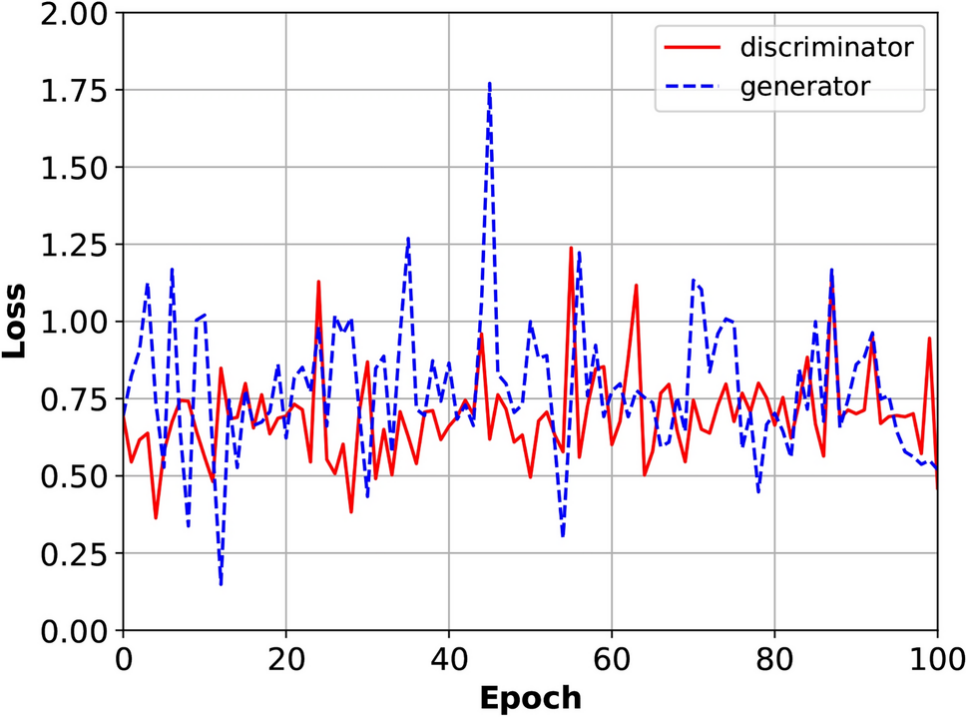
Loss diagram comparing the discriminator loss (red) and generator loss (blue) during the training of the GAN.

Ultimately, the loss behavior depicted in Fig. 3 underscores the GAN’s proficiency in capturing and replicating the intricate absorption properties of the metamaterial-based absorber. Unlike typical neural networks with monotonically decreasing loss, GANs rely on a dynamic adversarial framework, aiming for a Nash equilibrium where neither the generator nor the discriminator can unilaterally improve. This competitive interplay naturally results in fluctuating loss values, reflecting the network’s continuous process of mutual refinement and optimization. These oscillations are not indicative of instability but rather signify the GAN’s capacity to iteratively adapt and learn the complex mappings required for accurate inverse design.

As mentioned, this network is introduced to approximate the optimal design parameters for unit cells aimed at achieving absorbers with absorption rates exceeding 90%. The generated parameters undergo a filtering threshold, retaining only those with high absorption rates. In fact, parameters validated by the MLP neural network for absorption levels above the desired threshold are preserved; otherwise, the estimation process for a novel design iterates until the criteria are met. Once the optimal parameters are determined, they undergo precise validation using the CST software framework to ensure the accuracy of the neural network’s predictions.

### Design space and data extraction

The design space for data generation encompasses six dimensions, each corresponding to variable parameters of the unit cell. These parameters are denoted as $\textbf{P} = \{p_1, p_2, p_3, p_4, p_5, p_6\}$, depicted in Fig. 1. To construct a comprehensive dataset, absorbance values are extracted across 55 discrete frequencies within the 8 to 12 GHz range. These frequency samples are represented by $\textbf{F} = \{f_1, f_2, \ldots , f_{55}\}$.

In this regard, the absorption $A$ at a frequency of $f_i$ for a given set of parameters $\textbf{P}$ is defined as:

4 $$\begin{aligned} A(f_i, \textbf{P}) = 1 - |S_{11}(f_i, \textbf{P})|^2 - |S_{21}(f_i, \textbf{P})|^2. \end{aligned}$$

### Neural network architecture and training

The neural network, designed to optimize these six parameters, aims for absorption values exceeding 90% by applying a filter

after the output layer. Given the interpolation nature of the problem, a fully connected network architecture was selected, and its details are summarized in Table 2. By employing a deep neural network approach, the complexity of mapping between input and output data is managed effectively, with a preference for multiple layers over merely increasing the neuron count per layer.

**Table 2:** MLP neural network architecture.

Layer Type	Number of Units
Input Layer	6
Hidden Layer 1	1224
Hidden Layer 2	1224
Hidden Layer 3	512
Hidden Layer 4	256
Output Layer	55

**Activation function:** The network architecture integrates Rectified Linear Unit (ReLU) activation functions in the intermediate layers which described as $\text {ReLU}(x) = \max (0, x)$, and a Sigmoid activation function $\sigma (x) = \frac{1}{1 + \exp (-x)}$ in the output layer to handle the analog nature of the output values^32^.

**Optimization problem:** The training process utilizes the Mean Squared Error (MSE) loss function for optimization as follows:

5 $$\begin{aligned} \mathscr {L}_{\text {MSE}} = \frac{1}{n} \sum _{i=1}^{n} (y_i - \hat{y}_i)^2, \end{aligned}$$

where the Adam optimization algorithm^33^, with 20% of the data reserved for validation to continuously monitor network performance, is utilized. Early stopping conditions are implemented to fine-tune the network parameters and minimize validation loss, thereby preventing overfitting^34^.

In the initial phase of applying our intelligent approximation method, we employed this MLP neural network to predict the absorption characteristics of the designed absorbers. Trained on a curated dataset, utilizing Specificity Index (SI) and Specificity-Mutual Information (SMI) techniques to ensure high data quality for efficient training, this neural network demonstrated strong predictive capabilities, accurately matching absorption value predictions with actual data.

**Fig. 4:**
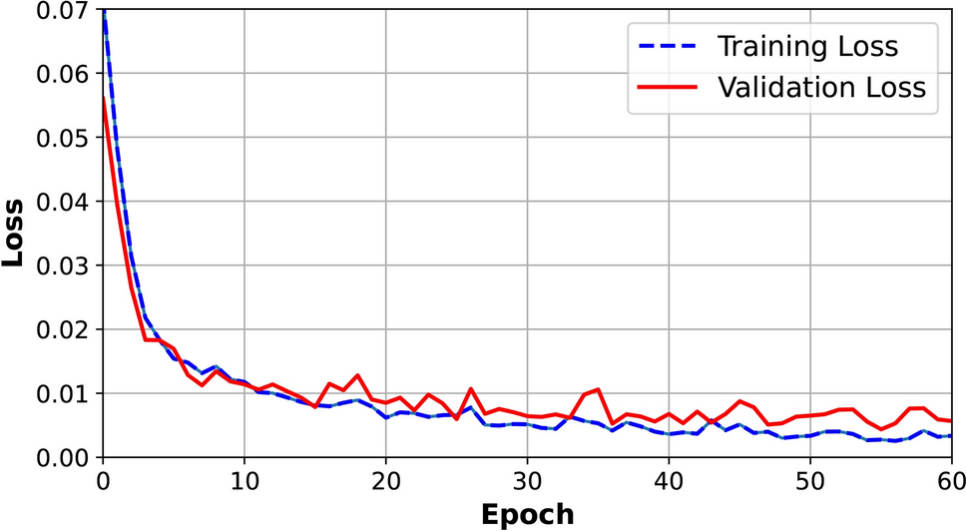
MSE loss of the multi-layer perceptron neural network during training and validation.

**Fig. 5:**
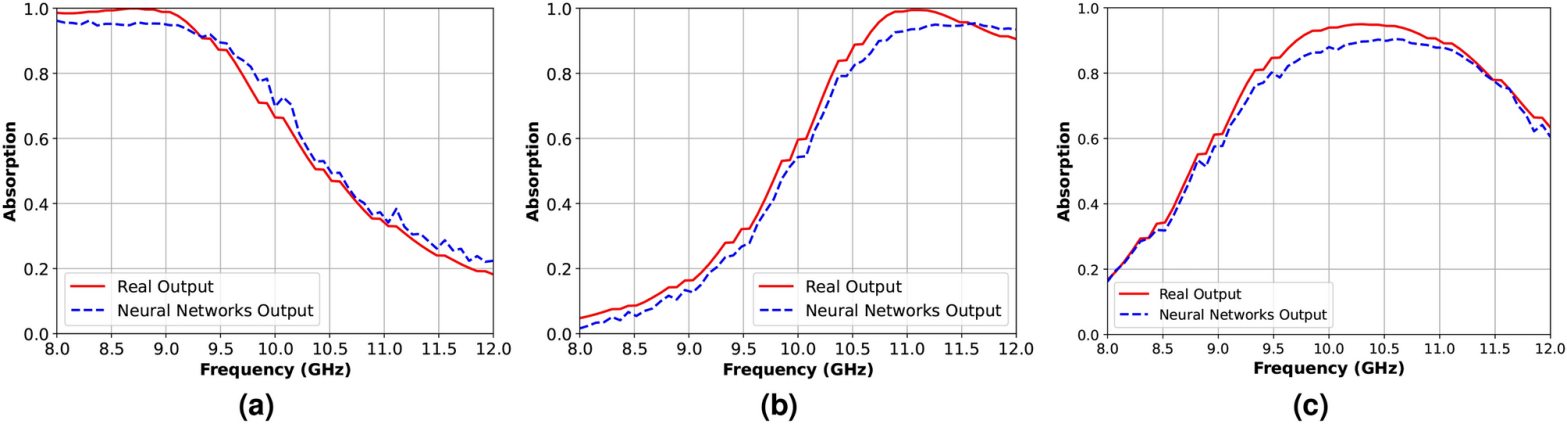
Comparison of absorption achieved from full-wave numerical simulation (solid red curves) performed by CST with the absorption predicted by the trained neural network (dashed blue curves).

By utilizing the collected data, the neural network converged with satisfactory MSE loss values of 0.0100 for training and 0.0174 for validation, as illustrated in Fig. 4. To further validate the network’s performance, in Fig. 5, we have compared the predicted results with the real-world outputs obtained from CST simulations for various input parameters shown in Table 3. As illustrated in this figure, the predictions of our trained network closely align with the ground truth values derived from CST, indicating its readiness for practical deployment in applications akin to our study. Consequently, this neural network effectively serves as a simulator, offering valuable insights into the absorption behavior of various absorber designs.

**Table 3:** Parameters for different subfigures in Fig. 5.

Subfigure	Parameters [$a, W_1, W_2, L_1, L_2, S_1$]
(a)	[6.9, 1.7, 1.9, 1.6, 3.8, 0.7]
(b)	[6.4, 3.9, 3.02, 0.7, 3.7, 0.2]
(c)	[4.8, 3.7, 3.7, 0.4, 2.4, 0.2]

## Results and discussion

**Fig. 6:**
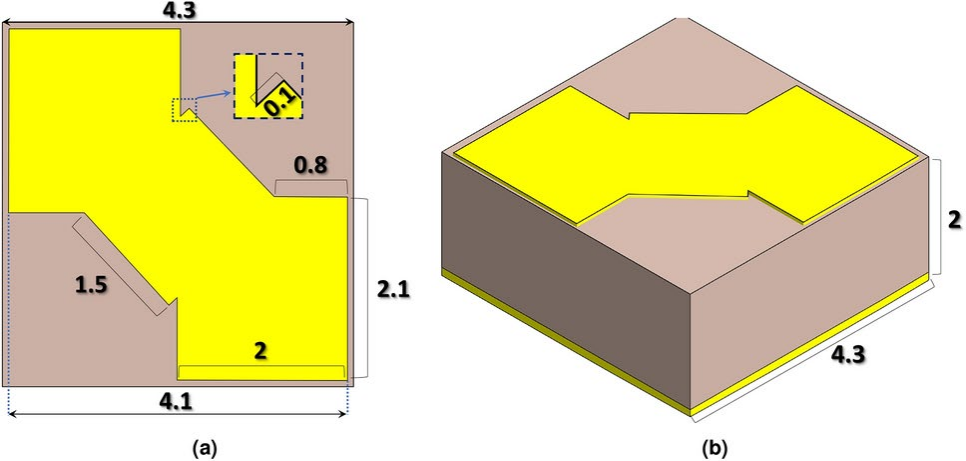
The final optimum design achieved using the proposed deep-learning-based methodology: (**a**) top view, (**b**) perspective view. Dimensions are in millimeters (mm).

In this section, we investigate the performance of the optimized unit cell designed using the proposed deep-learning-based methodology, both numerically and experimentally. The optimal design achieved is shown in Fig. 6, with parameters listed in Table 4.

**Table 4:** Optimized unit cell parameters achieved using the proposed deep learning-based architecture.

**Variable**	*a*	$W_1$	$W_2$	$L_1$	$L_2$	$S_1$	*h*
**Value (mm)**	4.3	2.07	1.76	2.14	1.5	0.1	2

**Fig. 7:**
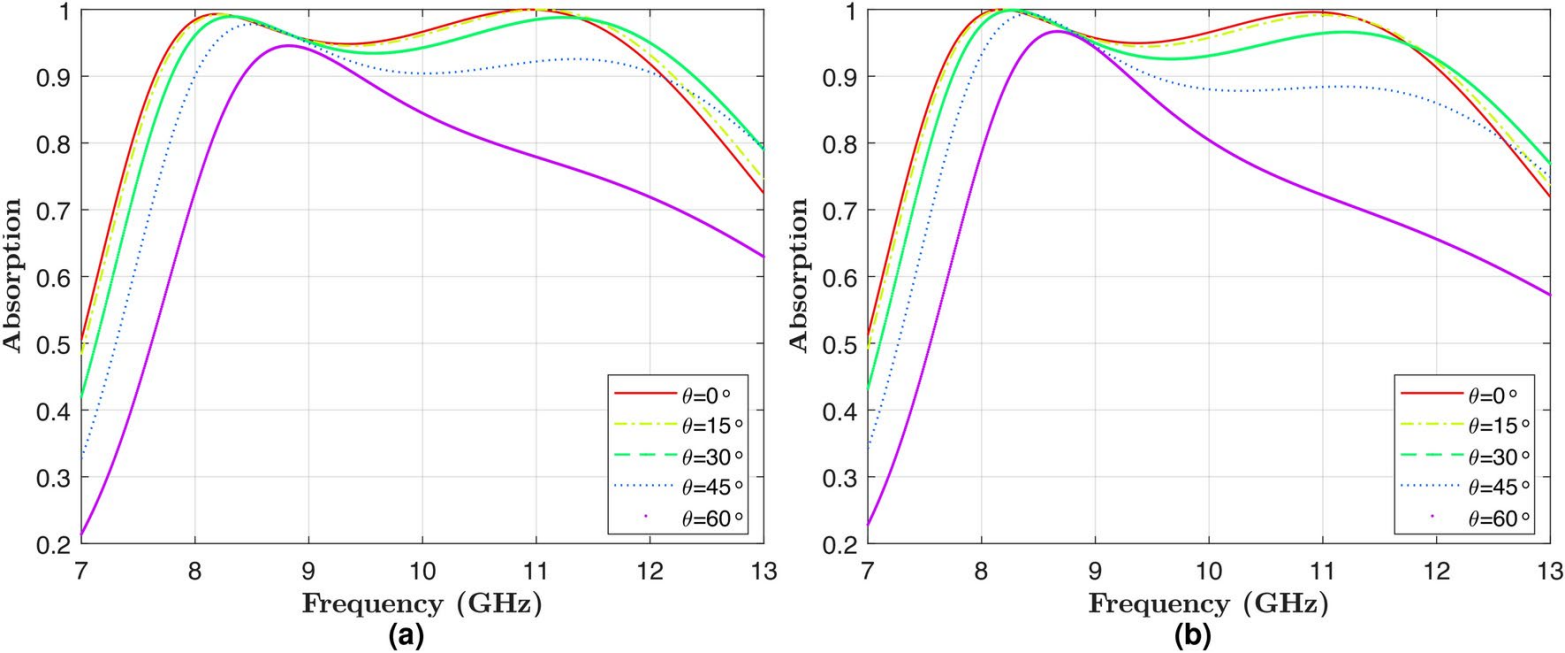
(**a**) Numerically calculated absorption for different incidence angles under TE polarization. (**b**) Numerically calculated absorption for different incidence angles under TM polarization.

**Fig. 8:**
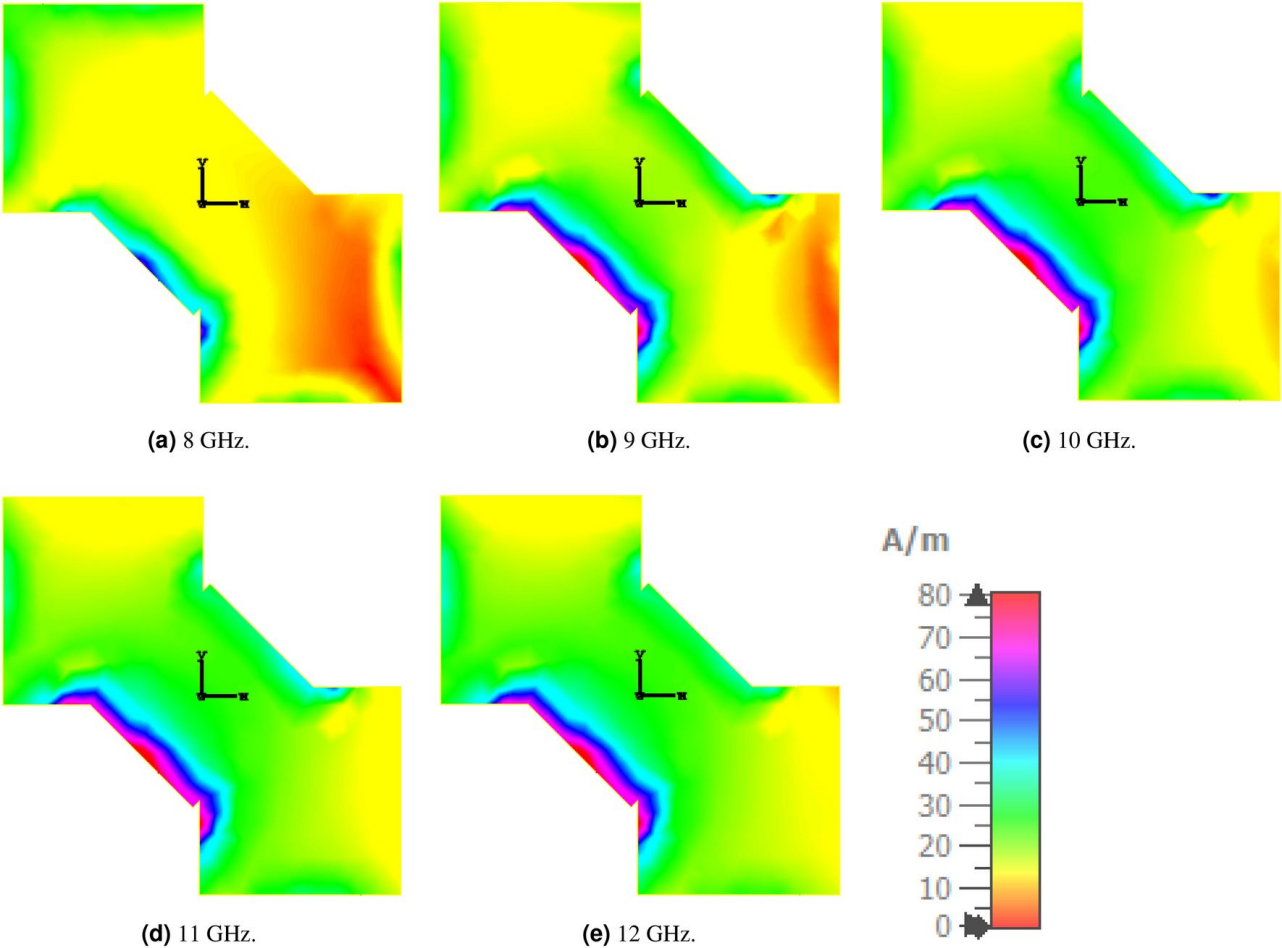
Numerically calculated surface current distribution on the optimized structure illustrating resonance at different frequencies.

To evaluate the performance of the optimized unit cell numerically, we conducted full-wave electromagnetic simulations using CST Microwave Studio. In these simulations, the optimized unit cell was illuminated by an EM plane wave, and the reflection and transmission coefficients of the cell were calculated numerically. Absorption was then calculated using Equation (1). The results of these simulations are shown in Figs. 7a-8.

Fig. 7a illustrates the absorption of the optimized unit cell versus operational frequency for different incident angles of the illuminating electromagnetic wave, with TE polarization. As shown, the absorption ratio exceeds 90% for all frequencies in the range of 8-12 GHz and for all incident angles from 0 to 45 degrees, indicating exceptional performance for the optimized structure. Additionally, results shown for a 60 degree incident angle reveal that while a slight reduction in absorption is observed, the design maintains robust performance, with absorption mostly exceeding 80% (Fig. 7a).

Similarly, Fig. 7b illustrates the absorption performance of the optimized unit cell as a function of frequency for different incidence angles under TM polarization. The results show that the absorption is close to 90% within the 8–12 GHz range for incidence angles ranging from 0 to 45 degrees, while a slight decrease at 45 degrees still presents a very good broadband performance. The absorption above 80% is maintained for an incidence angle of 60$^{\circ }$, where the most significant drop in absorption is at the frequency range of 10–12 GHz. This is due to the small impedance mismatch at oblique angles of incidence for TM polarization. However, the overall performance is highly efficient for a wide range of incidence angles. It represents that the absorber designed serves well for a wide incidence angle for both the TE and TM polarization states satisfactorily.

It is important to note that polarization conversion does occur in this design. However, this phenomenon does not substantially affect the performance for the intended applications, particularly in radar systems. The structural symmetry ensures consistent operation for both TE and TM polarization modes, which are predominantly encountered in typical radar scenarios. Additionally, the use of mixed or circular polarizations is generally rare and confined to specific niche applications.

To gain insight into the absorption mechanism of the proposed wideband absorber, the induced surface current distributions at various frequencies were analyzed and are depicted in Fig. 8. The figure reveals that, although the induced surface current is strong at all examined frequencies, its distribution across the cell varies. This suggests that the structure resonates at these frequencies with a high induced current, yet the resonance occurs at different parts of the cell providing a wide band response.

**Fig. 9:**
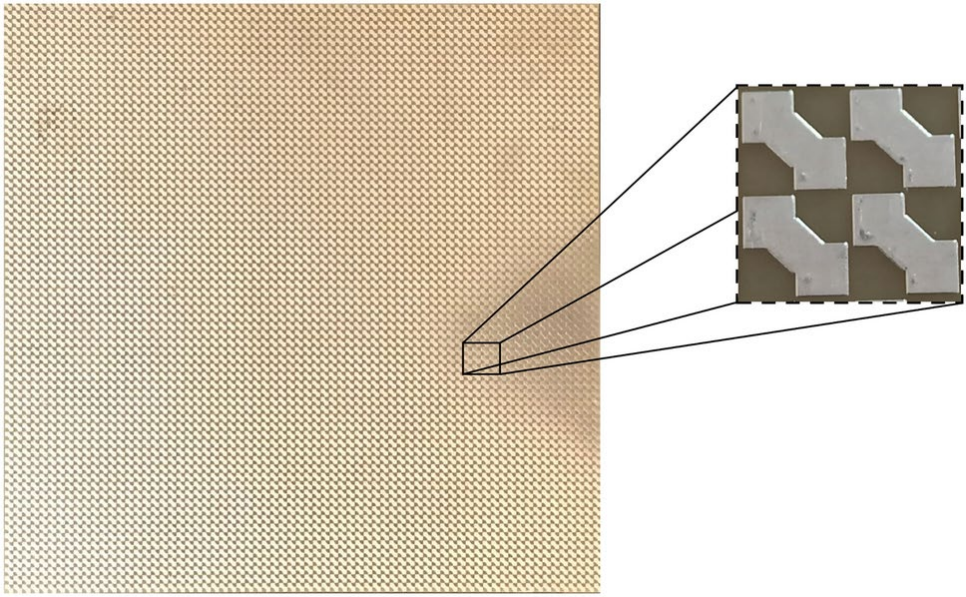
Fabricated prototype of the optimized design.

**Fig. 10:**
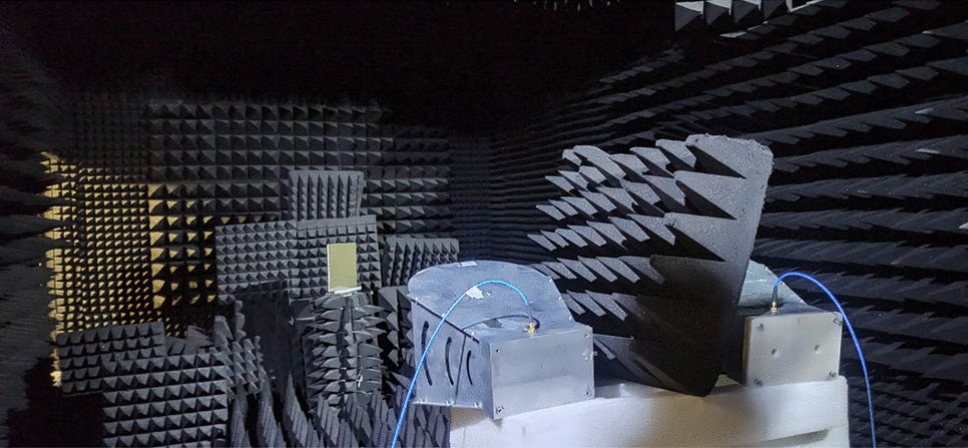
The experimental setup used for measurement of the reflection of the fabricated absorber.

To evaluate the performance of the optimized design, the structure was fabricated on a 2 mm FR4 PCB, as shown in Fig. 9. The measurements were conducted in a shielded anechoic chamber to minimize electromagnetic interference and unwanted reflections. The test setup consisted of a pair of horn antennas acting as transmitter and receiver, alongside a network analyzer (refer to Fig. 10). A metallic plate with the same dimensions as the fabricated absorber was used as a reference to measure the baseline reflection coefficient under identical conditions. The absorber was then measured, ensuring consistent alignment and distance relative to the antennas.

**Fig. 11:**
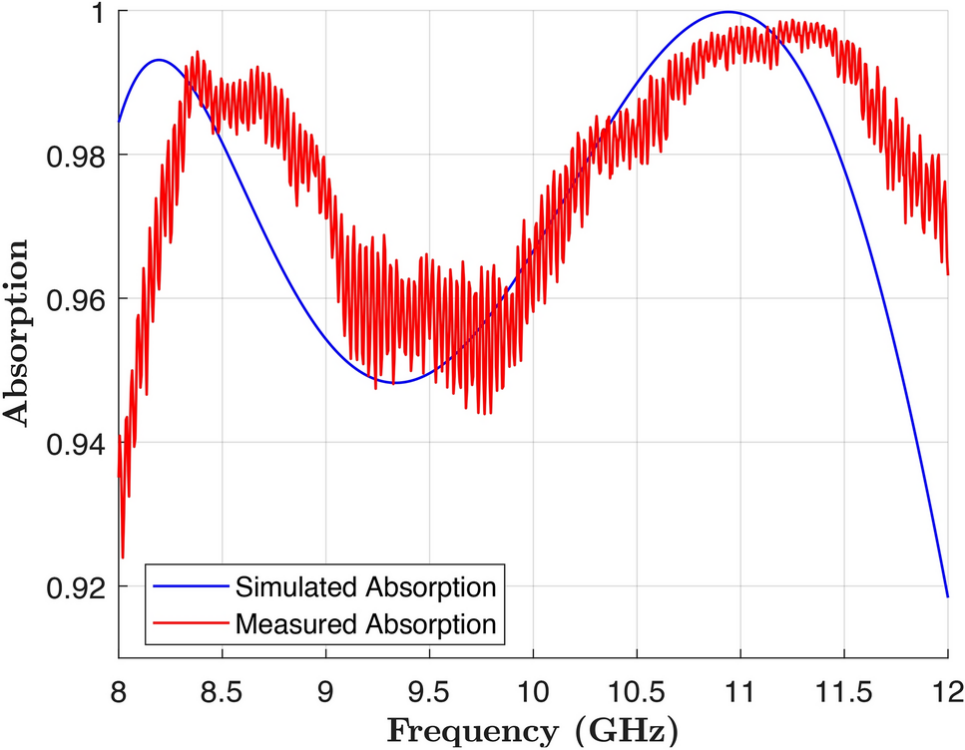
Comparison of the simulated and measured results.

This setup ensured accurate and repeatable measurements, with the results compared to numerical simulations, as shown in Fig. 11. The figure compares the experimental data with full-wave simulation results. The measurements show that the optimized structure achieves over 90% absorption across the entire frequency range of 8–12 GHz, confirming the wideband response of the design. While the experimental and numerical results are in general agreement, a slight frequency shift is observed. This shift is attributed to fabrication tolerances, such as minor deviations in the dimensions of the unit cells, and variations in the FR4 substrate’s thickness and dielectric properties ($\varepsilon _r$ and $\tan \delta$). Additionally, experimental uncertainties, such as absorber misalignment in the setup, environmental interferences, and minor variations in the angle of incidence, may have contributed to the frequency shift. In contrast, the numerical simulations assume ideal conditions, including a perfectly periodic structure and uniform substrate properties, which do not account for these practical variabilities.

Table 5 compares this work with recently published studies on metamaterial-based EM absorbers in terms of frequency bandwidth, thickness, sensitivity to the angle of incidence, and method of realization. As illustrated in this table, works realized through PCB fabrication^5,7,35,36^ either operate at several individual frequencies rather than covering a frequency range, thus being considered multi-band instead of wide-band^5,7^, or suffer from very high thickness^35,36^. For example, the absorber proposed in^7^ operates at individual frequencies of 2.45, 4, and 5.8 GHz without covering a frequency range. Similarly, the absorber presented in^35^, although covering a wide frequency range of 3-12 GHz, has a high thickness of 9.2 mm. The absorber presented in^18^ has a wide operational bandwidth and relatively small thickness; however, its realization requires lumped elements soldered onto the PCB, making the fabrication complex and unsuitable for mass production. This also causes the structure to be fragile due to the high number of resistors involved. Therefore, Comparing this work with previously reported absorbers realized through PCB fabrication, our design offers the advantage of wide bandwidth and low thickness while avoiding complex fabrication processes such as using lumped resistors. On the other hand, metamaterial-based absorbers designed for realization through 3D printing technology^37,38^ provide wide bandwidth results, as shown in Table 5, but at the cost of high absorber thickness. Furthermore, compared to PCB fabrication, 3D printing technology is more expensive and time-consuming. By using water in 3D-printed absorbers, researchers have shown that the thickness can be reduced^39,40^; however, absorbers with water inside are not suitable for many practical applications as the water needs to be replaced regularly, and it can also result in corrosion, decreasing the absorber’s lifetime. In conclusion, Table 5 clearly demonstrates that the structure proposed in this work offers significant advantages over previously reported metamaterial-based EM absorbers. It is wide-band, thin, and both easy and inexpensive to fabricate.

**Table 5:** Comparison of this work with previously reported metamaterial-based electromagnetic absorbers.

Reference	Thickness (mm)	Range of Incident Angles (Absorption Ratio)	Frequency of Operation (GHz)	Method of Realization
^35^	9.2	0-30$^{\circ }$ ($>90$%)	3–12	PCB
^5^	2	0-60$^{\circ }$ ($>98$%)	8.49, 9.87, 10.65, 12.24	PCB
^7^	3.7	0-30$^{\circ }$ ($>80$%)	2.45, 4.0, 5.8	PCB
^36^	9	0-30$^{\circ }$ ($>90$%)	1.24–3.14	PCB
^18^	3.25	0-45$^{\circ }$ ($>90$%)	10–20	Lumped element soldered on PCB
^37^	5.9	0-40$^{\circ }$ ($>90$%)	6.5–50.7	3D printing
^38^	5.1	0-45$^{\circ }$ ($>90$%)	16.52–100	3D printing
^39^	3.6	0-30$^{\circ }$ ($>80$%)	9.3–49.0	Water-filled channels realized by 3D printers
^40^	4.8	0-40$^{\circ }$ ($>70$%)	10.4–30	Water-filled channels realized by 3D printers
This work	2	0-45$^{\circ }$ ($>90$%)	8–12	PCB

## Conclusions

A deep learning methodology for the design and optimization of wideband metamaterial-based absorbers was introduced and verified both numerically and experimentally. This approach employs a GAN for parameter estimation and design generation, alongside a MLP network for accurate absorption value prediction. The results demonstrated that this framework effectively optimized absorber designs with high precision and reduced computational time. The optimal design’s performance was validated both numerically and experimentally, confirming high absorption across all frequencies in the range of 8-12 GHz and for all incident angles between 0 and 45 degrees, for both TE and TM polarizations of the incident electromagnetic wave.

## Methods

The data required for training the MLP neural network was generated using numerical simulations in CST Microwave Studio. These simulations were conducted in the frequency range of 8-12 GHz, utilizing the Floquet port for excitation and periodic boundary conditions to emulate the behavior of an infinite array. The reflection coefficient ( $S_{11}$) was extracted from the numerical simulations, and the absorption was calculated based on this reflection coefficient.

The fabricated optimal absorber was tested in a controlled environment using two horn antennas and a network analyzer. One horn antenna was used to illuminate the absorber with the incident wave, while the other was used to receive the reflected wave. Both antennas were connected to the network analyzer, enabling S-parameter measurements. The absorption was then calculated using the measured S-parameters.

## Data Availability

The datasets generated and/or analyzed during the current study are available from the corresponding author upon reasonable request.
